# Dr. Andrew B. Stevens

**Published:** 1906-05

**Authors:** 


Dr. Andrew B. Stevens, a graduate of the University of Buffalo, 1873, died at his home at Watertown, N. Y., April 7, 1906, aged 66 years. He was a civil war veteran, one of the older physicians of Watertown, and his death was due to disease of the heart.

